# Supplementary material for: Heat adaptation of phage T7 under an extended genetic code
Source: Virus Evol. 2021 Dec 1;7(2):veab100. doi: 10.1093/ve/veab100 (PMC8923235; doi:10.1093/ve/veab100)
Supplement: veab100_Supp [file veab100_supp.zip › Sequencing_Analysis.zip!/Sequencing_Analysis/S1/marginal.html]

BRESEQ :: Marginal Predictions


|  |  |
| --- | --- |
|  | ***breseq***  version 0.30.1    mutation predictions \| marginal predictions \| summary statistics \| genome diff \| command line log |

| Marginal read alignment evidence | | | | | | | | | | | |
| --- | --- | --- | --- | --- | --- | --- | --- | --- | --- | --- | --- |
|  | seq id | position | | ref | new | freq | score (cons/poly) | reads | annotation | genes | product |
| \* | NC\_001604.1 | 26,148 | 0 | A | G | 79.4% | 6777.8 / inf | 4179 | intergenic (–/–) | *–/–* | –/– |
| \* | NC\_001604.1 | 35,366 | 0 | C | A | 77.2% | 5753.9 / inf | 4301 | intergenic (–/–) | *–/–* | –/– |
| \* | NC\_001604.1 | 29,697 | 0 | C | T | 72.4% | 4495.6 / inf | 3542 | intergenic (–/–) | *–/–* | –/– |
| \* | NC\_001604.1 | 34,968 | 0 | C | A | 64.9% | 2014.2 / inf | 3835 | intergenic (–/–) | *–/–* | –/– |
| \* | NC\_001604.1 | 21,285 | 0 | A | C | 57.1% | 3354.1 / inf | 4479 | intergenic (–/–) | *–/–* | –/– |
| \* | NC\_001604.1 | 36,121 | 0 | G | A | 54.0% | 1070.6 / inf | 5292 | intergenic (–/–) | *–/–* | –/– |
| \* | NC\_001604.1 | 39,930 | 1 | . | C | 44.4% | 3.8 / 13.5 | 9 | intergenic (–/–) | *–/–* | –/– |
| \* | NC\_001604.1 | 20,544 | 0 | G | A | 38.4% | 3029.7 / inf | 4188 | intergenic (–/–) | *–/–* | –/– |
| \* | NC\_001604.1 | 14,339 | 0 | T | C | 34.2% | 3953.0 / inf | 4528 | intergenic (–/–) | *–/–* | –/– |
| \* | NC\_001604.1 | 13,566 | 1 | . | A | 33.4% | 5269.3 / inf | 4545 | intergenic (–/–) | *–/–* | –/– |
| \* | NC\_001604.1 | 35,266 | 0 | A | G | 33.3% | 3896.1 / inf | 4112 | intergenic (–/–) | *–/–* | –/– |

| Marginal new junction evidence (lowest skew 10 of 22 shown) | | | | | | | | | | | |
| --- | --- | --- | --- | --- | --- | --- | --- | --- | --- | --- | --- |
|  | | seq id | position | reads (cov) | reads (cov) | score | skew | freq | annotation | gene | product |
| \* | ? | NC\_001604.1 | = 16604 | 1296 (0.310) | 133 (0.030) | 92/590 | 18.3 | 8.0% | intergenic (–/–) | *–/–* | –/– |
|  | ? | NC\_001604.1 | 17028 = | 1786 (0.430) |  |  |  |  | intergenic (–/–) | *–/–* | –/– |
| \* | ? | NC\_001604.1 | = 1256 | 0 (0.000) | 154 (0.040) | 67/574 | 22.6 | 99.7% | intergenic (–/–) | *–/–* | –/– |
|  | ? | NC\_001604.1 | 2737 = | 1 (0.000) |  |  |  |  | intergenic (–/–) | *–/–* | –/– |
| \* | ? | NC\_001604.1 | = 7972 | 5047 (1.200) | 75 (0.020) | 61/588 | 24.5 | 1.5% | intergenic (–/–) | *–/–* | –/– |
|  | ? | NC\_001604.1 | 8524 = | 4926 (1.200) |  |  |  |  | intergenic (–/–) | *–/–* | –/– |
| \* | ? | NC\_001604.1 | = 9895 | 4719 (1.120) | 64 (0.020) | 49/592 | 27.8 | 1.3% | intergenic (–/–) | *–/–* | –/– |
|  | ? | NC\_001604.1 | 10215 = | 4867 (1.180) |  |  |  |  | intergenic (–/–) | *–/–* | –/– |
| \* | ? | NC\_001604.1 | = 13578 | 10 (0.000) | 121 (0.030) | 37/584 | 31.3 | 93.5% | intergenic (–/–) | *–/–* | –/– |
|  | ? | NC\_001604.1 | 14348 = | 7 (0.000) |  |  |  |  | intergenic (–/–) | *–/–* | –/– |
| \* | ? | NC\_001604.1 | = 281 | 3834 (1.030) | 908 (0.230) +TAAAGACCAGACC | 18/508 | 36.9 | 18.1% | intergenic (–/–) | *–/–* | –/– |
|  | ? | NC\_001604.1 | 282 = | 4288 (1.020) |  |  |  |  | intergenic (–/–) | *–/–* | –/– |
| \* | ? | NC\_001604.1 | = 13586 | 3 (0.000) | 50 (0.010) | 23/592 | 37.3 | 30.3% | intergenic (–/–) | *–/–* | –/– |
|  | ? | NC\_001604.1 | 14356 = | 227 (0.050) |  |  |  |  | intergenic (–/–) | *–/–* | –/– |
| \* | ? | NC\_001604.1 | 295 = | 5273 (1.260) | 27 (0.010) | 15/560 | 40.5 | 0.54% | intergenic (–/–) | *–/–* | –/– |
|  | ? | NC\_001604.1 | = 281 | 4936 (1.260) |  |  |  |  | intergenic (–/–) | *–/–* | –/– |
| \* | ? | NC\_001604.1 | 111 = | 2696 (0.640) | 18 (0.000) | 12/592 | 43.6 | 0.66% | intergenic (–/–) | *–/–* | –/– |
|  | ? | NC\_001604.1 | 121 = | 2794 (0.670) |  |  |  |  | intergenic (–/–) | *–/–* | –/– |
| \* | ? | NC\_001604.1 | = 19635 | 4193 (1.000) | 12 (0.000) | 11/590 | 44.2 | 0.29% | intergenic (–/–) | *–/–* | –/– |
|  | ? | NC\_001604.1 | 19645 = | 4138 (1.000) |  |  |  |  | intergenic (–/–) | *–/–* | –/– |
